# Comorbidity Burden, Polypharmacy and Xerostomia Severity in Institutionalized Older Adults

**DOI:** 10.3390/medicina62071377

**Published:** 2026-07-17

**Authors:** Mădălina Monica Bicheru, Andreea Zamfirescu, Elena Preoteasa, Adrian Iustin Georgevici, Nicolae Vladimir Bicheru, Cristina Teodora Preoteasa

**Affiliations:** 1 Department of Prosthodontics, Faculty of Dentistry, “Carol Davila” University of Medicine and Pharmacy, 020021 Bucharest, Romania; 2 Department of Geriatrics and Gerontology, Faculty of Midwifery and Nursing, “Carol Davila” University of Medicine and Pharmacy, 050474 Bucharest, Romania; 3 Katholisches Klinikum, Ruhr-University Bochum, 44801 Bochum, Germany; 4 Department of Scientific Research Methods-Ergonomics, Faculty of Dentistry, “Carol Davila” University of Medicine and Pharmacy, 020021 Bucharest, Romania

**Keywords:** xerostomia, hyposalivation, polypharmacy, multimorbidity, complete denture, institutionalized older adults, oral health

## Abstract

*Background and Objectives:* Xerostomia is common among institutionalized older adults, but the relative contributions of comorbidity burden and medication-class burden remain incompletely clarified. This study evaluated their associations with Xerostomia Inventory (XI) severity and explored related salivary dysfunction and clinical oral health findings. *Materials and Methods:* We conducted a cross-sectional study of 90 institutionalized older adults in Bucharest, Romania. Xerostomia Inventory (XI) scores, a binary dry-mouth question, chairside salivary assessments, comorbidity categories, medication classes, oral mucosal findings, and complete denture retention and stability were recorded. Primary analyses used hierarchical regression and bootstrap statistical decomposition; secondary and exploratory analyses evaluated salivary concordance, medication classes, denture-related findings, and oral mucosal conditions. *Results:* Medication-class count remained associated with XI severity after adjustment for comorbidity burden (partial r = 0.445, *p* < 0.001), whereas the association between comorbidity burden and XI was no longer significant after adjustment for medication-class count (partial r = −0.063, *p* = 0.557). Inclusion of medication-class count increased the explained variance in XI scores (ΔR^2^ = 0.253, *p* < 0.001). Bootstrap decomposition yielded similar findings, with a significant indirect estimate and a non-significant direct estimate. Among complete denture wearers, XI severity was associated with maxillary denture instability (ρ = −0.687, n = 44). *Conclusions:* In this cohort, medication-class burden showed the strongest association with xerostomia severity, whereas comorbidity burden was not independently associated after adjustment. Given the cross-sectional design, these findings should be interpreted as exploratory associations and support future prospective studies integrating medication review with geriatric-prosthodontic assessment.

## 1. Introduction

Xerostomia is one of the most frequent oral complaints in institutionalized older adults, with reported prevalence ranging from 20% to 72% depending on population characteristics and diagnostic criteria [1,2]. In residential care settings, dry mouth often coexists with multimorbidity, intensive medication use, impaired salivary function, and oral prosthodontic problems [3,4]. Xerogenic medications are well recognized as contributors to dry mouth [5,6], but in institutionalized denture-wearing older adults, it remains unclear whether xerostomia severity is more closely associated with comorbidities, the polypharmacy that often accompanies it, or both. However, the relative contribution of medication burden versus comorbidity burden remains insufficiently defined in institutionalized older adults, particularly in those with edentulism and removable dentures.

In older adults, multimorbidity often leads to therapeutic accumulation, and the resulting number of medication classes may affect salivary gland function through several mechanisms, including anticholinergic effects, altered autonomic regulation, and central nervous system depression [7,8]. If the association between comorbidities and xerostomia severity is attenuated after accounting for the number of medication classes, clinical attention may shift from pathophysiology alone toward medication review as one potentially modifiable component of care [3,9].

The issue is particularly relevant in edentulous or partially edentulous older adults wearing removable dentures. In this group, saliva is not only a biological fluid involved in lubrication and oral protection, but also a functional component of denture retention, stability, and comfort [10,11,12]. Reduced salivary flow or altered salivary quality may therefore intensify symptoms that are perceived as xerostomia while simultaneously worsening prosthodontic performance.

The Xerostomia Inventory (XI) is an 11-item instrument that captures dry mouth severity on a continuous scale, ranging from 11 to 55 [13]. Unlike a binary screening question [14], it offers the resolution needed to detect graded symptom burden and to examine individual symptoms separately [15]. This matters in older denture wearers because some symptoms, such as difficulty eating or swallowing, may reflect prosthodontic dysfunction as well as salivary impairment, whereas oral stickiness may be more closely linked to glandular dysfunction [10,16].

The primary aim of this study was to evaluate the association between comorbidity burden, medication-class count, and xerostomia severity, while controlling for age and sex. Secondarily, we explored the concordance between subjective xerostomia and salivary dysfunction, as well as the associations of specific medication classes with xerostomia severity, complete denture retention and stability.

## 2. Materials and Methods

### 2.1. Study Design and Population

This cross-sectional study enrolled 90 institutionalized older adults from social care centres for dependent persons in Bucharest, Romania, between September 2025 and February 2026. All residents meeting the eligibility criteria during the study period were invited to participate. The final sample size was determined by the number of eligible residents available in the participating institutions and by consent to participate; therefore, a convenience sample was used. No formal a priori sample size calculation was performed because the study was exploratory and was conducted in a restricted institutional population. Inclusion criteria were age ≥ 60 years, complete edentulism in at least one jaw, and capacity to cooperate with questionnaires and clinical examination. Individuals with significant communication or comprehension difficulties were excluded. The study was approved by the Ethics Committee of the “Carol Davila” University of Medicine and Pharmacy, Bucharest, Romania (approval No. 22332/05.09.2025), and written informed consent was obtained from all participants. To reduce potential information bias, standardized questionnaires and predefined clinical assessment protocols were used for all participants. Medical history and medication data were verified against institutional medical records whenever possible. Nevertheless, selection bias may have occurred because participation was limited to residents able to provide informed consent and complete the study procedures. Data were collected through structured interview, questionnaire administration, intraoral clinical examination performed by a dentist, and consultation of each participant’s medical file. All intraoral and prosthodontic assessments were performed by the same dentist using predefined examination criteria. Data recording was performed during the examination under the examiner’s supervision. Sociodemographic and behavioural variables included age, sex, education level, and current smoking status. Prosthodontic status variables included edentulism pattern and removable denture use. This study was reported in accordance with the Strengthening the Reporting of Observational Studies in Epidemiology (STROBE) guidelines. The completed STROBE checklist is provided as Supplementary Table S1.

### 2.2. Clinical Assessments

Health conditions were documented from medical records and classified into 13 categories (cardiovascular, endocrine, neurological, psychiatric, gastrointestinal, respiratory, renal, dermatological, musculoskeletal, ophthalmological, urogenital, granulomatous, viral infections). Comorbidity burden was defined as the count of these 13 categories. A category-based approach was used to provide a standardized measure of multimorbidity and to avoid overrepresentation of closely related diagnoses recorded in institutional medical files.

Polypharmacy was defined as ≥5 concurrently prescribed medication classes [17]. The administration of at least one anxiolytic, sedative or hypnotic, antidepressant, antiepileptic, or antiparkinsonian medication class was grouped into a composite variable termed “any central nervous system (CNS) medication”. This exploratory variable was created because these medication classes may influence xerostomia through central nervous system effects, sedation, autonomic modulation, or associated xerogenic effects.

Subjective xerostomia was assessed both as a binary screening question (“do you have a sensation of dry mouth?”) and also the Xerostomia Inventory (XI-11), an 11-item instrument scored on a 5-point Likert scale (1 = never to 5 = very frequently), with higher scores indicating greater severity [13].

Following Villa et al. [9], an unstimulated salivary flow (USF) specimen was collected over 5 min (passive drool, mouth at rest), and flow was classified by the examining clinician as low or normal. USF low therefore refers to a chairside binary resting-saliva classification rather than a laboratory flow rate. Five chairside salivary parameters were graded ordinally using the GC Saliva Check Buffer kit per kit instructions [18]: mucosal hydration (1–2), saliva viscosity (1–3), pH (1–3), stimulated flow rate (1–3), and buffer capacity (1–3), with lower scores indicating greater impairment. Objective hyposalivation was operationalized as a stimulated flow rate ≤ 2 on the GC ordinal scale, namely the “very low” or “low” categories. This GC variable is therefore an ordinal saliva-parameter classification, not a continuous flow-rate measure.

Denture retention and stability were evaluated using the CU-modified Kapur index, with maxillary retention scored 0–3 and stability scored 0–2. Dentures were classified as acceptable or unacceptable. Oral mucosal manifestations were documented: denture-related lesions (decubitus), fibrous hyperplasia, stomatitis (classified by Newton grade I–III [19]), angular cheilitis, atrophic glossitis, coated tongue, and caries on remaining teeth.

### 2.3. Statistical Analysis

We evaluated the association between comorbidity count and XI score, adjusted for age and sex, using nonparametric bootstrap regression with 1000 bootstrap resamples and BCa confidence intervals. We used a mediation-type statistical decomposition to examine the role of medication-class count in the comorbidity–xerostomia association. Since the cross-sectional design strictly precludes causal inference, the algorithm’s outputs are defined throughout this manuscript as the Indirect Estimate (IE) and Direct Estimate (DE) and interpreted as statistical associations, not causal effects.

A hierarchical multivariable regression of XI on demographics, comorbidity burden, and recorded medication-class count provided a transparent supporting model for the same primary question. The anticholinergic cognitive burden score was computed per Boustani and colleagues [20]. In an additional model step, anxiolytic use was entered separately to explore whether a medication class previously associated with xerostomia contributed information beyond overall medication-class burden.

Secondary analyses tested the medication-count association using partial correlations adjusting for age, sex, and comorbidity burden, and examined concordance between subjective xerostomia and salivary dysfunction using Cohen’s kappa, exact McNemar testing, sensitivity, specificity, positive predictive value, and negative predictive value.

Exploratory analyses used Spearman rank correlations for ordinal XI, Kapur, salivary, and item-level variables, and Mann–Whitney U tests with rank-biserial r for individual medication-class and oral-mucosal comparisons. Holm correction was used for primary hypotheses and Benjamini–Hochberg correction within each exploratory family; no correction was applied across the primary-to-exploratory boundary.

Denture-use and Kapur-adjusted models were treated as clinical-context sensitivity checks because these variables may lie downstream of salivary dysfunction rather than act as core confounders.

Analyses were performed in R 4.5 [21] with two-sided 95% confidence intervals and α = 0.05.

Missing data were limited to selected medication-class variables used in exploratory analyses. No imputation procedures were performed. Analyses involving these variables were conducted using available-case (complete-case) analysis; therefore, sample sizes may vary across individual comparisons.

## 3. Results

### 3.1. Participants

During the study period, 113 residents were assessed for eligibility. Fifteen were excluded: five did not meet the age criterion (<60 years), four had no complete edentulism in either jaw, and six had significant communication or comprehension difficulties. Of the 98 eligible residents, eight declined participation. The remaining 90 consented, were enrolled, and completed the clinical examination.

The 90 participants had a mean age of 79.5 years (SD 7.1; range 64 to 89), and 50 (55.6%) were female. The education level was primary school in 48 (53.3%), secondary in 41 (45.6%), and university in 1. Current smoking was reported by 27 participants (30%). All participants had total maxillary edentulism; 5 (5.6%) had partial mandibular edentulism. Removable dentures were present in 44 participants (48.9%).

The mean number of comorbidity categories was 7.4 (SD 2.2; range 3 to 13), and the mean number of medication classes was 8.9 (SD 3.3; range 2 to 16). Polypharmacy (≥5 medication classes [17]) was present in 82 participants (91.1%) (Table 1).

The mean XI score was 31.7 (SD 9.2; median 33; IQR 9). In our cohort, the participants with an XI score below 28 declared no subjective dry mouth.

Bivariate correlations showed a weak comorbidity-XI association (ρ = 0.202, 95% CI [−0.005, 0.393], *p* = 0.056) and a moderate recorded medication-class-count-XI association (ρ = 0.477, 95% CI [0.299, 0.623], *p* < 0.001). When recorded medication-class count was held constant, the comorbidity-XI association was no longer statistically significant (partial r = −0.063, 95% CI [−0.268, 0.147], *p* = 0.557); when comorbidity burden was held constant, the recorded medication-class-count-XI association remained significant (partial r = 0.445, 95% CI [0.260, 0.598], *p* < 0.001).

**Figure 1:**
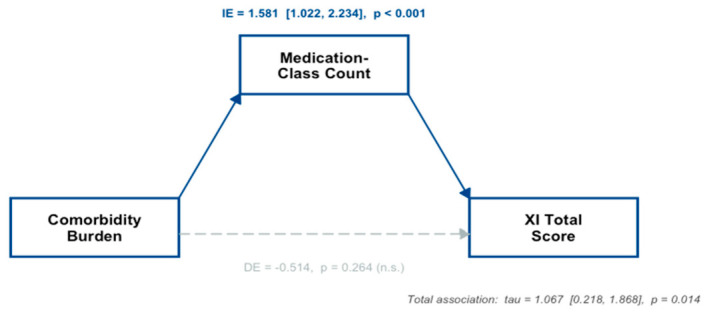
Statistical decomposition of the cross-sectional comorbidity–xerostomia association. Solid arrows = indirect statistical association; dashed arrow = direct statistical association. IE = Indirect Estimate; DE = Direct Estimate; τ = total association. Values are unstandardized coefficients with 95% BCa bootstrap confidence intervals (1000 resamples). Outputs should be interpreted as statistical associations given the cross-sectional design and do not imply causality.

Bootstrap decomposition showed that the total statistical association of comorbidity burden with XI was 1.067 (95% CI [0.218, 1.868], *p* = 0.014). The Indirect Estimate (IE) through recorded medication-class count was 1.581 (95% CI [1.022, 2.234], *p* < 0.001), whereas the Direct Estimate (DE) was not statistically significant (DE = −0.514, 95% CI [−1.486, 0.463], *p* = 0.264). Hierarchical regression showed the same ordering: adding recorded medication-class count to demographics and comorbidity burden increased explained XI variance (R^2^ = 0.345, ΔR^2^ = 0.253, *p* < 0.001). The statistical decomposition and hierarchical regression are summarized in Figure 1 and Table 2.

### 3.2. Medication Classes

**Figure 2:**
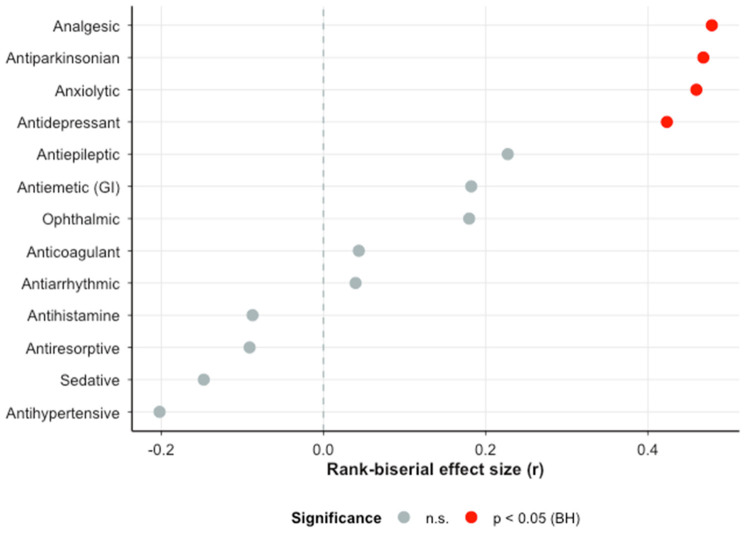
Effect of individual medication classes on XI scores. Points show rank-biserial effect sizes (r); red = significant after Benjamini–Hochberg correction (*p* < 0.05), grey = non-significant (n.s.).

We explored whether the association with the number of medication classes could be explained as a total anticholinergic effect from multiple drug classes. However, this analysis remained non-significant and was not superior to the simple count of medication classes (partial r = 0.004, *p* = 0.98). The largest exploratory finding was the concurrent use of “any CNS medication” (anxiolytic, sedative, hypnotic, antidepressant, antiepileptic, or antiparkinsonian). Among the 79 residents receiving at least one such class, the median XI was 34; among the 11 not receiving any such class, it was 17 (effect size r = 0.886, *p* < 0.001). Per-class effects are shown in Table 3 and Figure 2.

**Table 3:** Effect of individual medication classes on Xerostomia Inventory scores. Effect size r = rank-biserial correlation from Mann–Whitney U test. *p* (BH) = Benjamini–Hochberg-adjusted two-sided *p*-value. Bold rows indicate *p* (BH) < 0.05. n for individual classes varies due to missing data.

Medication Class	N	Median XI (Absent)	Median XI (Present)	Effect Size (r)	*p* (BH)
Analgesic	54	29.0	34.0	0.479	<0.001
Anxiolytic	48	30.0	34.0	0.460	<0.001
Antiparkinsonian	26	30.5	34.0	0.468	0.003
Antidepressant	58	29.0	34.0	0.423	0.003
Antiepileptic	32	31.5	33.0	0.227	0.22
Antiemetic (GI)	56	32.0	33.5	0.182	0.28
Ophthalmic	33	32.0	34.0	0.180	0.28
Antihypertensive	76	35.5	33.0	−0.202	0.36
Sedative	45	36.0	34.0	−0.148	0.43
Antiresorptive	32	33.0	32.5	−0.091	0.61
Antihistamine	26	35.0	33.0	−0.087	0.64
Anticoagulant	33	33.0	34.0	0.044	0.75
Antiarrhythmic	36	33.0	33.0	0.040	0.75

### 3.3. Denture Retention, Stability, and Oral Mucosal Findings

Among the 44 denture wearers, Kapur maxillary denture stability correlated inversely with XI (ρ = −0.687, 95% CI [−0.817, −0.490], *p* < 0.001). “Sticky mouth” showed the strongest correlation with maxillary retention (ρ = −0.778, 95% CI [−0.873, −0.626], *p* < 0.001), while “difficulty swallowing” and “dry mouth when eating” were also correlated with retention (ρ = −0.602 and −0.522, respectively, both *p* < 0.001).

The total count of oral pathologies correlated strongly with XI (ρ = 0.636, 95% CI [0.494, 0.745], *p* < 0.001). Individual associations were observed for stomatitis (ρ = 0.673, *p* < 0.001) and decubitus lesions (ρ = 0.626, *p* < 0.001). Denture plaque was associated with stomatitis (OR = 4.29, 95% CI [1.14, 20.25], Fisher *p* = 0.021).

### 3.4. Concordance with Salivary Dysfunction

In this cohort, XI ≥ 28, the binary xerostomia question, and the clinician-classified low unstimulated salivary flow identified the same 69 participants. For the concordance analyses, these concordant measures were therefore treated as a single xerostomia classification. Against objective hyposalivation (GC kit stimulated flow ≤ 2), the xerostomia classification showed substantial agreement (Cohen’s kappa = 0.689); 80 of 90 residents were concordant, including 5 false positives and 5 false negatives; the exact McNemar test was non-significant (*p* = 1.0; Table 4).

## 4. Discussion

In this cross-sectional institutional cohort, the number of medication classes showed the strongest observed association with XI severity. The association between comorbidities and XI was weak in bivariate analysis and not statistically significant after controlling for the number of medication classes. Conversely, the association between medication-class count and XI remained significant after adjustment for comorbidity. Our findings are consistent with previous reports linking xerostomia to polypharmacy in older populations, although causality remains uncertain given the cross-sectional design and potential residual confounding [5,6,7,22].

These findings should be interpreted within the broader geriatric symptom-burden context of institutionalized older adults, in whom comorbidity burden, medication-class burden, salivary dysfunction, and removable denture use frequently coexist. Previous geriatric studies have similarly shown that multimorbidity, psychological status, and functional impairment are closely interconnected in older institutionalized patients, supporting the concept that xerostomia may reflect a broader multidimensional vulnerability profile rather than an isolated oral symptom [23]. Previous work in older adults with multimorbidity has shown that dry mouth may occur as part of a multidimensional symptom profile rather than as an isolated complaint, with multiple concurrent symptoms frequently reported in this population [24]. In our cohort, xerostomia severity may therefore reflect the overlap between systemic disease accumulation, therapeutic exposure, impaired oral lubrication, and local prosthodontic or mucosal conditions. The attenuation of the comorbidity–XI association after accounting for medication-class count suggests that medication burden may partly capture the clinical complexity accompanying multimorbidity. At the same time, the associations between XI severity, maxillary denture stability, retention-related symptoms, and oral mucosal findings support the clinical relevance of xerostomia beyond symptom reporting alone.

The statistical decomposition indicates that XI was associated with the number of medication classes, whereas the direct association between the number of comorbidities and XI was non-significant. One of the main findings is that, after adjustment for medication-class count, no other variable showed a significant direct association with XI. However, the interactions could be more subtle than our analysis and sample size revealed. From a clinical point of view, multiple other factors may influence salivation, including age-related hypodipsia, blunted dryness perception, or symptom-reporting competition in the most multimorbid residents.

Medication class count may also capture cumulative systemic burden, drug–drug interactions, and non-anticholinergic xerogenic effects. In this population with severe polypharmacy (median 9.0 medication classes), a simple class count may capture both cumulative exposure and their additional synergies or interactions. Our interpretation differs from that of Desoutter and colleagues [22], who found that only anticholinergic class membership predicted xerostomia in geriatric ward patients. This discrepancy may reflect methodological differences, particularly their drug-level anticholinergic scoring compared with our class-level proxy [20]. Therefore, medication-class count should be interpreted as a pragmatic marker of therapeutic burden rather than as a substitute for detailed drug-level xerogenic or anticholinergic assessment.

Beyond the pharmacological explanation, our findings illustrate how saliva acts as both a structural element in denture wearers and a mucosal protectant. Saliva lubricates the mucosa under the denture and helps maintain stability and retention [10,11,12], so reduced flow plausibly amplifies symptoms of stickiness and difficulty swallowing. In our cohort, “sticky mouth” was closely associated with maxillary denture retention (ρ = −0.778), a finding consistent with the saliva thin-film physics described by O’Brien [11,16]. The same loss of lubrication and protection increases susceptibility to frictional trauma, soreness, and inflammation at the denture–mucosa interface; oral-pathology count correlated strongly with XI (ρ = 0.636), and the denture–plaque–stomatitis association (OR = 4.29) is clinically compatible with plaque-associated denture inflammation [25]. Decreased masticatory efficiency and lower oral health-related quality of life in completely edentulous subjects have likewise been linked to denture instability [26].

From a practical point of view, these findings support considering integrated geriatric-prosthodontic assessment in institutionalized older adults wearing removable dentures, while intervention effects still require prospective evaluation. The administration of CNS medication could invite the clinician to review the exposure to other anticholinergic medication, especially when the patient reports subjectively dry mouth. Medication review, together with denture-fit assessment, evaluation of retention and stability, and oral mucosal monitoring, may represent a useful approach when managing institutionalized older adults with xerostomia, although its effectiveness requires confirmation in prospective studies [25]. The clinical significance of xerostomia in this population is not confined to symptom reporting: it may also signal compromised prosthodontic status and increased risk of denture-related complications [26,27]. Recent evidence also suggests that digital health and e-health approaches may improve monitoring and long-term management of chronic conditions in older adults, potentially facilitating multidisciplinary follow-up and medication review in patients with xerostomia and multimorbidity [28]. Prospective multi-centre designs are needed to evaluate whether systematic medication review and integrated prosthodontic care reduce xerostomia severity.

Potential sources of bias should also be considered when interpreting these findings. Because participants were recruited exclusively from institutional care facilities, the observed prevalence of multimorbidity, polypharmacy, and xerostomia may be higher than that seen in community-dwelling older adults. Conversely, exclusion of individuals with significant communication or comprehension difficulties may have resulted in underrepresentation of the most frail residents. The direction and magnitude of these potential biases cannot be quantified but should be considered when interpreting the reported associations.

Several limitations should be considered when interpreting these findings. First, the cross-sectional design does not allow confirmation of temporal sequence, and the statistical decomposition should be interpreted as a decomposition of associations rather than as evidence of causality. Second, the sample size of 90 participants and the use of a convenience sample form two institutions limit the complexity of the statistical models and the generalizability of the findings. Third, medication exposure was recorded at the level of medication classes rather than individual compounds, doses, treatment duration, or cumulative exposure, because of the structure of the institutional medical records. Therefore, medication class count should be interpreted as a pragmatic proxy for therapeutic burden rather than as a drug-level xerogenic or anticholinergic score. Fourth, salivary parameters were graded using chairside ordinal or categorical classifications, which preclude direct comparison with laboratory-based continuous salivary flow measurements. Finally, all participants had complete maxillary edentulism and were recruited from only two institutional care facilities in Bucharest. Consequently, the findings may not be directly generalizable to community-dwelling older adults, healthier elderly populations, individuals with different prosthodontic status, or institutionalized populations in other healthcare systems. Replication in larger and more diverse cohorts is therefore needed. Prospective multicentre longitudinal studies are needed to clarify the temporal relationship among multimorbidity, prescribing patterns, salivary dysfunction, denture-related factors and xerostomia symptoms.

## 5. Conclusions

In institutionalized older adults, the number of administered medication classes was associated with xerostomia severity. However, after controlling for other variables, comorbidity burden was not independently associated with xerostomia in our cohort. These exploratory findings should not be interpreted as evidence of causality. These findings suggest that medication review may be clinically relevant in institutionalized older adults reporting subjective dry mouth. Symptom reporting alone may not fully reflect the complexity of xerostomia in this population, and future studies should further investigate the potential contribution of denture-related and oral mucosal factors.

## Figures and Tables

**Table 1:** Participant characteristics by xerostomia status (N = 90). Continuous variables are mean (SD); categorical variables are n (%).

Characteristic	Overall N = 90	Without Xerostomia N = 21	Xerostomia N = 69	*p*-Value ^1^
Age (years), Mean (SD)	80 (7)	78 (8)	80 (7)	0.37
Sex, n (%)				0.74
Female	50 (56%)	11 (52%)	39 (57%)	
Male	40 (44%)	10 (48%)	30 (43%)	
Education, n (%)				>0.99
Primary	48 (53%)	11 (52%)	37 (54%)	
Secondary	41 (46%)	10 (48%)	31 (45%)	
University	1 (1.1%)	0 (0%)	1 (1.4%)	
Current smoker, n (%)	27 (30%)	5 (24%)	22 (32%)	0.48
Comorbidity categories, Mean (SD)	7 (2)	6 (2)	8 (2)	0.019
Medication classes, Mean (SD)	9 (3)	5 (2)	10 (3)	<0.001
Polypharmacy, n (%)	82 (91%)	13 (62%)	69 (100%)	<0.001
Denture use, n (%)	44 (49%)	10 (48%)	34 (49%)	0.89

^1^ Wilcoxon rank sum test; Pearson’s Chi-squared test; Fisher’s exact test.

**Table 2:** Hierarchical regression predicting Xerostomia Inventory total score. Adjusted R-squared (final model) = 0.350; n = 90.

Step	R^2^	ΔR^2^	F-Test *p* (vs. Prev.)
1. Demographics (age, sex)	0.037		
2. +Comorbidity burden	0.092	0.055	0.026
3. +Medication-class count	0.345	0.253	<0.001
4. +Anxiolytic use	0.387	0.042	0.018

**Table 4:** Concordance between the unified xerostomia classification (XI ≥ 28, equivalent to the binary screen and to USF classified as low; kappa = 1.0 for each pair) and objective hyposalivation defined as GC kit stimulated flow ≤ 2.

Subjective Classification	Hyposalivation Present	Hyposalivation Absent	Total
Positive	64	5	69
Negative	5	16	21
Total	69	21	90

Sensitivity 0.928 [0.841–0.969]; Specificity 0.762 [0.549–0.894]; Cohen’s kappa = 0.689 [0.511–0.868]; exact McNemar *p* = 1.0.

## Data Availability

The dataset analyzed during the current study is available from the corresponding authors upon reasonable request, in accordance with privacy and ethical restrictions. Analytic code is maintained in a private project repository and can be shared on request for academic review.

## Supplementary Materials

The following supporting information can be downloaded at https://www.mdpi.com/article/10.3390/medicina62071377/s1, Table S1: STROBE Checklist for Cross-Sectional Studies.
