# Supplementary material for: Comorbidity Burden, Polypharmacy and Xerostomia Severity in Institutionalized Older Adults
Source: Medicina (Kaunas). 2026 Jul 17;62(7):1377. doi: 10.3390/medicina62071377 (PMC13414273; doi:10.3390/medicina62071377)
Supplement: Supplementary file 1 [file medicina-62-01377-s001.zip › medicina-4369023-supplementary.pdf]

Supplementary Table S1

STROBE Checklist for Cross-Sectional Studies

Manuscript Title: Comorbidity Burden, Polypharmacy and Xerostomia Severity in Institutionalized Older Adults

This study was reported in accordance with the STROBE statement for cross-sectional studies.

Item	Description	Location
1a Title	Cross-sectional study of recorded medication-class burden and xerostomia in institutionalized older adults	Title
1b Abstract	Structured abstract with associational framing	Abstract
2 Background/rationale	Multimorbidity, medication-class burden, xerostomia in institutionalized older adults	Introduction
3 Objectives	Exploratory aims; associational analyses only	Introduction
4 Study design	Cross-sectional	Methods
5 Setting	Bucharest residential social-care facility, Sep 2025-Feb 2026	Methods
6 Participants	Inclusion/exclusion criteria, consent procedure	Methods and Results
7 Variables	XI, comorbidity count, recorded medication-class count, Kapur, salivary kit	Methods
8 Data sources/measurement	Medical records, structured interview, intraoral examination	Methods
9 Bias	Convenience sample; legacy chart limitations; Romanian XI translation	Methods and Limitations
10 Study size	Sample size and power discussion	Methods and Limitations
11 Quantitative variables	Continuous and ordinal handling described	Methods
12a Statistical methods (primary)	Hierarchical regression and statistical decomposition as primary	Methods and Results
12b Statistical methods (subgroups)	Medication-count audit, comorbidity definitions, adjustment checks, CNS and concordance sensitivity	Methods
13 Participants	Eligibility, refusals, enrolment counts	Results
14 Descriptive data	Demographics by xerostomia status	Results and Table 1
15 Outcome data	XI distribution	Results
16 Main results	Statistical decomposition, hierarchical regression, medication-class effects	Results, Tables 2-4, Figures 1-3
17 Other analyses	Item-level XI, oral pathology, Kapur, robustness tables	Results
18 Key results	Medication-count association with sensitivity caveats	Discussion

Item	Description	Location
19 Limitations	Cross-sectional design; class-level proxy; count-definition sensitivity;	Limitations
20 Interpretation	Associational framing only; clinical implications motivate prospective work	Discussion and Conclusions
21 Generalisability	Institutionalized cohort, edentulous focus limits external generalisability	Limitations
22 Funding	Listed in main manuscript	Funding and declarations
